# Extrachromosomal DNA Length and Antibiograms of *Staphylococcus aureus* and *Pseudomonas aeruginosa* Isolated from Tears of HIV/AIDS Patients after Curing with Sodium Dodecyl Sulphate

**DOI:** 10.5539/gjhs.v4n1p229

**Published:** 2012-01-01

**Authors:** Ajayi B. O., Otajevwo F. D.

**Affiliations:** Department of Optometry Faculty of Life Sciences University of Benin, Nigeria; Dept of Microbiology & Biotechnology Western Delta University Oghara, Nigeria

**Keywords:** Extrachromosomal, DNA, Antibiogram, *Staph aureus*, *P. aeruginosa*, Sodium dodecyl sulphate

## Abstract

*Staphylococcus aureus* and *Pseudomonas aeruginosa* strains were isolated from eye swab samples randomly obtained from 100 seropositive HIV/AIDS patients who reported to various anti-retroviral treatment clinics at the University of Benin Teaching Hospital and Central Hospital both based in Benin City, Nigeria. Invitro antibiotic sensitivity patterns of strains before curing were determined by the Kirby-Bauer disc diffusion technique. Resistance plasmid DNA of multidrug resistant strains was cured with 0.1% sodium dodecyl sulphate and cured strains were again subjected to invitro antibiotic sensitivity testing. EcoRI and Hind III restriction endonuclease enzymes were used to make cuts on extracted plasmid DNA whose length sizes were then determined. A total of 36 (36.0%) strains made up of 27 (75.0%) *Staphylococcus. aureus* and 9 (25.0%) *Pseudomonas aeruginosa* were isolated of which 7 (19.4%) strains showed multidrug resistance to ciprofloxacin, pefloxacin, ofloxacine, gentamycin, tetracycline, ampicillin, chloramphenicol, nitrofurantoin and erythromycin. All seven multidrug resistant strains before curing, recorded 85.7%, 42.9%, 14.3% and 14.3% sensitivity in that decreasing order to ciprofloxacin, pefloxacin, ofloxacin and gentamycin respectively. There was 0.0% sensitivity each to tetracycline and ampicillin. After curing, there was enhanced sensitivity of 100.0%, 85.7%, 28.6% and 71.4% respectively. There was also 28.6% and 57.1% improved sensitivity to tetracycline and ampicillin after curing. Before curing, there was 76.2% average resistance to all used antibiotics and this reduced to 47.6% after curing *Staph. aureus* plasmid DNA. In the case of *Pseudomonas aeruginosa*, there was an average resistance of 76.3% before curing which fell to 42.5% after curing. EcoRI restriction enzyme gave the plasmid DNA length of *Staphylococcus aureus* strain 04 as 4.0Kb and this size depended upon the distance between recognition sites. Isolation of 36 (36.0%) strains of both isolates from 100 eye swabs shows the danger these organisms portend to all categories of opticians. The cheapness and high sensitivity of gentamycin justifies its use as eye drops for treatment of some eye infections. Curing of plasmid DNA is an indication that if SDS is administered to the organisms in sublethal doses, it can lead to the elimination of plasmid DNA without adverse effect on the genomic DNA of the bacterial strains.

## 1. Introduction

AIDS nearly always affects the eyes and ophthalmic signs (symptoms) were the initial signs (among others), that led to the diagnosis of HIV infection in its terminal stage (Cheesborough, 1990). Fujikawa *et al*. (1985) reported that when patients contact HIV, the virus can infect nearly every ocular tissue as well as the tear (lachrymal) gland. They found human T-cell lymphotrophic virus (HTLV-III) in tears.

*Staphylococcus aureus* and *Psuedomonas aeruginosa* are opportunistic pathogens in humans and animals and are one of the frequent sources of hospital and community acquired infections. They can infect the eyes through contaminated fingers and contact lens. Contact lens also causes abrasion of ocular structure thereby increasing the chances of the “wearers” developing ocular infection. Disinfecting systems are important part of infection control practices and aid in the prevention of infection.

*Pseudomonas aeruginosa*, among other diseases, causes eye infections such as keratitis and neonatal ophthalmia. *Pseudomonas aeruginosa* can colonize the ocular epithelium by means of a fimbrial attachment to sialic acid receptors. If the defense of the environment is compromised in any way, the bacterium can proliferate rapidly and through the production of enzymes such as elastase, alkaline protease as well as exotoxins which can cause rapidly destructive infections which may eventually lead to loss of the entire eye (Alejandro *et al.*, 2002).

Capriotti *et al*. (2008) in their study of normal flora in 276 conjunctival swabs of healthy eyes of a rural population in Sierra Leone, isolated coagulase negative *Staph aureus* (28.6%), coagulase positive *Staph aureus* (19.9%), *Haemophilus* spp (9.8%) and *Norcadia* spp (6.5%). Grasbon *et al*. (1995) examined 100 conjunctival swabs for the prevalence of coagulase negative *Staph aureus* and isolated 151 different strains of coagulase positive *Staph aureus* in 86 samples while coagulase negative *Staphylococci* accounted for the remainder.

Maria *et al*. (2000) worked on 40 conjunctival swabs of health professionals to know the conjunctival microflora of clinically healthy persons who work in the hospital environment. They isolated *Staph. epidermidis* (45.0%), *Bacillus* spp (29.0%), *Proteus* spp (61.0%), *Citrobacter* spp (2.1%), *Moraxella* spp (2.1%) and *Proteus mirabilis* (2.1%). Yasuyuki *et al*. (2005) in a study to compare the conjunctival flora of HIV seropositive and seronegative patients found no difference between the conjunctival flora of HIV positive and negative patients

Most bacterial strains are lysogenic and many of the toxins and products of these strains are mediated by plasmids which play a major role chemically as mediators of antimicrobial resistance (Alejandro *et al.*, 2002). Griffith (1986) reported that antibiotic resistance plasmids were molecules of circular DNA containing an aONA segment called the replication region which allows the plasmid to propagate itself independently of the machinery that reproduces the chromosomal DNA.

Aminocyclotol resistance plasmid was isolated and characterized in *Staphylococcus aureus* by Gray (1999). According to him, the plasmid was divided into two interrelated groups due to loss or gain of defined DNA sequences. Also, Stiffer *et al*. (1974) isolated and characterized Kanamycin resistance plasmid form *Staph. aureus*. They reported that the plasmid had a molecular weight of 9.2 × 104 Daltons.

A restriction endonuclease functions by scanning the length of a DNA molecule. Once it encounters its particular specific recognition sequence, it binds to the DNA molecules and makes one cut in each of the two sugar-phosphate backbones of the double helix. The positions of these two cuts both in relation to each other and to the recognition sequence itself are determined by the identity of the restriction endonuclease used to cleave the molecule in the first place.

Lofdahi *et al*. (1978) characterized small molecular weight plasmids from *Staph. aureus* with respect to size, restriction endonuclease pattern and transforming capacity. The plasmids p5194 and pCM4 which encode streptomycin and chloramphenicol resistance respectively contained 3.0 and 2.0 megadalton of DNA. Both plasmids transformed *Staph. aureus* with high efficacy. Whereas pC 194 contained only one average size for endonuclease Hind II, pS194 contained single cleavage for endonuclease Hind III and EcoRI.

The multidrug resistance nature of some organisms such as *Pseudomonas aeruginosa* and *Staphylococcus aureus* (among others), found in seropositive HIV/AIDS patients have posed a serious problem in the effective management of eye infections suffered by these patients from time to time. This study therefore, is aimed at determining the “extrachrosomal DNA length and antibiograms of *Staphylococcus aureus* and *Pseudomonas aeruginosa* isolated from tears of HIV/AIDS patients after curing with Sodium Dodecyl Sulphate” with the following objectives:

1) Isolate *Staph. aureus* and *Pseudomonas aeruginosa* from eye swabs of HIV/AIDS patients.
2) Determine the sensitivity patterns (antibiograms) of these isolates to some selected antibiotics.
3) Cure the isolates of their plasmid DNA using sodium dodecyl sulphate (SDS).
4) Use restriction endonucleases (EcoRI and Hind III) to cut and determine the length of the isolates plasmid DNA.

## 2. Materials and Methods

### 2.1 Sampling

With informed consent and approval obtained from the ethical committee of the hospital managements, eye swabs of 100 seropositive HIV/AIDS patients receiving treatment at the medical wards of the University of Benin Teaching Hospital (UBTH) and the Central Hospital both based in Benin City, Nigeria were randomly selected and obtained for the study. Collection of samples was done without consideration for age, sex, profession and family background. The seropositive HIV/AIDS status of patients used was as confirmed by the Enzyme Linked Immunosorbent Assay (ELISA) technique.

### 2.2 Processing of Samples

Obtained eye swabs were taken to the laboratory immediately for processing. Swabs were collected in duplicates per patient. While one was used for gram staining, the other was used for culture. Swabs were aseptically cultured on sterile MacConkey, Blood and Mannitol salt agar plates and incubated aerobically at 37°C for 24hours.

Isolates were identified culturally, morphologically, biochemically and by sugar fermentation according to schemes provided by Cowan and Steel (1993) and Cullimoore (2000). All catalase positive, coagulase positive colonies, gram positive cocci in clusters, glucose positive, mannitol positive (characteristic of *Staphylococcus aureus)* and all citrate positive, oxidase positive colonies with gray-greenish pigmentation, short gram negative rods in singles (characteristic of *Pseudomonas aeruginosa*) were stocked on agar slants for further use. Before curing, the strains were subjected to invitro antibiotic testing.

### 2.3 Invitro Antibiotic Testing

This was done according to the modified Bauer and Kirby (1997) disc diffusion technique. Sterile Nutrient agar plates and peptone water were prepared and dispensed into bijou bottles in accordance with manufacturer’s instructions. An inoculum of the stock culture of each strain was subcultured into sterile peptone water bijou bottles and incubated on the bench for 2-3hours. A sterile peptone water bottle (which was not subcultured) was used as control. All steps above were carried out with proper labellings. Sterile Nutrient agar plates were arranged and labeled for the strains of each isolate and one as control. The plates were flooded with the liquid culture in the bijou bottles and the control bijou bottle was equally used to flood the control plate. The excess liquid culture was drained off the plates. Using well sterilized pair of forceps, commercially obtained gram positive and gram negative multidrug discs was impregnated on flooded agar surface. While gram positive discs was used for *Staph. aureus* strains, gram negative discs were used for *Pseudomonas aeruginosa* strains. The control plate was divided into two halves. On one half, gram positive discs were impregnated and gram negative discs on the other half. All plates were then incubated aerobically at 37°C for 24hours. Results were interpreted according to the National Committee for Clinical Laboratory Standards (1997). Strains that were resistant to the drugs used invitro were subjected to curing with sodium dodecyl sulphate (SDS).

### 2.4 Plasmid Curing

Plasmid curing of *Staphylococcus aureus* and *Pseudomonas aeruginosa* strains that were resistant (did not show any zone of inhibition after incubation) was done according to the method described by Tomoeda *et al*. (1968). To 90ml of Nutrient broth, 10g of sodium dodecyl sulphate (SDS) was added. The resulting suspension was autoclaved, steamed for 1 hour, pH adjusted to 7.6 and then used as stock solution. Overnight broth culture of the multidrug resistant strains of *Staph. aureus* and *Pseudomonas aeruginosa* was each diluted 100 fold (0.1ml of broth + 9.9ml sterile water). To fresh 30ml of Nutrient broth, 0.5ml of diluted broth was added and mixture maintained at pH 7.6. This was incubated for 2 hours and 1% SDS stock solution was added and then incubated for up to 72hours (3days).

### 2.5 Invitro Antibiotic sensitivity testing of cured strains

Plasmid cured strains were again subjected to antibiotic sensitivity testing according to the method of Kirby-Bauer already described above.

### 2.6 Isolation of Plasmid DNA

Cured plasmid DNA of multidrug resistant strains of *Staph. aureus* and *Pseudomonas aeruginosa* was isolated by the alkaline phosphate method of Birmborn and Doly (1979).

### 2.7 Restriction Endonuclease Activity

Commercially available restriction endonucleases - EcoRI and Hind III were obtained and used to determine the sizes of short cuts on the isolated Plasmid DNA. With these sizes known, the entire plasmid DNA length was then determined. According to the method described by Esiobu (2008), 0.51 microlitre (0.51µl) of restriction endonucleases of EcoRI and Hind III each, was added to 1 microlitre of 1µg/ml of extracted DNA in a tube and mixed. The mixture was centrifuged for few seconds in a microcentrifuge and incubated at 37°C for 2hours. Ten microlitres of the mixture was taken and put in another tube and this was loaded into 1% Agarose gel stained with ethidium bromide. The electrophoretic tank was filled with 1XTBE. Agarose gel was also loaded with 5µl of extracted (uncut) genomic DNA. Gel was then run at 120 volts for 45mins and was visualized on a trans-illuminator at long UV range. The gel results were then analyzed to determine length of extracted (cured) DNA.

## 3. Results

Out of the 100 samples processed, 36(36.0%) strains made up of 27(75.0%) *Staphylococcus aureus* and 9(25.0%) *Pseudomonas aeruginosa* strains were obtained of which 3(11.1%) *Staph. aureus* and 4(44.4%) *Pseudomonas aeruginosa* strains showed multidrug resistance (Table 1).

**Table 1:** Occurrence of multidrug resistant strains among samples processed.

Isolates	No of strains isolated	No of strains showing multidrug resistance
*Staphylococcus aureus*	27 (75.0%)	3 (11.1%)
*Pseudomonas aeruginosa*	9 (25.0%)	4 (44.4%)
**Total**	**36 (100.0%)**	7

Before curing, 3(11.1%) *Staph.aureus* strains showed resistance to three or more antibiotics invitro while 4(44.4%) *Pseudomonas aeruginosa* strains showed resistance to three or more (multidrug resistance). After curing, 2(7.4%) *Staph. aureus* strains showed resistance to more than three antibiotics invitro while 2 (22.2%) *Pseudomonas aeruginosa* strains showed resistance to three or more antibiotics (Table 2).

**Table 2:** Invitro Antibiotic Susceptibility Patterns of Isolates strains before and after curing

Code of strains	Organisms	Antibiotics									
			CIP	PEF	OF	GN	TE	AMP	C	N	E
04	*Staph. aureus*	Before	S	R	R	R	R	R	NA	NA	R
		After	S	S	R	R	R	R	NA	NA	S
06	*Staph. aureus*	Before	S	S	R	R	R	R	NA	NA	S
		After	S	S	R	S	R	S	NA	NA	S
10	*Staph. aureus*	Before	S	R	R	R	R	R	NA	NA	R
		After	S	R	R	S	R	R	NA	NA	R
11	*Pseud. aeruginosa*	Before	R	R	S	S	R	R	R	R	NA
		After	S	S	S	R	S	R	R	R	
12	*Pseud. aeruginosa*	Before	S	S	R	R	R	R	S	S	NA
		After	S	S	S	S	R	S	R	S	
13	*Pseud. aeruginosa*	Before	S	R	R	R	R	R	S	S	NA
		After	S	S	R	S	S	S	R	S	
14	*Pseud. aeruginosa*	Before	S	S	R	R	R	R	R	S	NA
		After	S	S	R	S	R	S	R	S	
Total % sensitivity		Before	**85.7%**	**42.9%**	**14.3%**	**14.3%**	**0.0%**	**0.0%**			
		After	**100%**	**85.7%**	**28.6%**	**71.4%**	**28.6%**	**57.1%**			

After curing, *Staphylococcus aureus* didn’t show any sensitivity change to ciprocin as all strains remained sensitive before and after treatment with 0.1% sodium dodecyl sulphate. There was 33.3% reduction in resistance to pefloxacin after treatment. Similarly, there were 66.7%, 66.7% and 33.3% changes (or reduction) in resistance to gentamycin, ampicillin and erythromycin respectively after treatment or curing with SDS. However, all strains remained resistant to ofloxacin and tetracycline before and after curing (Tables 3 and 4). On average, there was 28.6% reduction in resistance to all the antibiotics after curing.

**Table 3:** Summary of Resistance pattern of Staph. aureus after curing with 0.1% Sodium Dodecyl Sulphate

Antibiotic	Before	After	% Change (Reduction) in Resistance
Ciprofloxacin	0%	0%	0%
Pefloxacine	66.7%	33.3%	33.3%
Ofloxacine	100.0%	100.0%	0.0%
Gentamycin	100%	33.3%	66.7%
Tetracycline	100.0%	100.0%	0.0%
Ampicillin	100%	33.3%	66.7%
Erythromycin	66.7%	33.3%	33.3%
**Average**	**76.2%**	**47.6%**	**28.6%**

**Table 4:** Summary of Resistance Pattern of *Pseudomonas aeruginosa* after curing with 0.1% SDS

Antibiotic	Before	After	% change (Reduction) in Resistance
Ciprofloxacin	75.0%	50.0%	25.0%
Pefloxacine	50.0%	0.0%	50.0%
Ofloxacine	75.0%	25.0%	50.0%
Gentamycin	75.0%	25.0%	50.0%
Ampicillin	100.0%	25.0%	75.0%
Nitrofurantoin	75.0%	75.0%	0.0%
Chloramphenicol	100.0%	100.0%	0.0%
Tetracycline	60.0%	40.0%	20.0%
**Average**	**76.3%**	**42.5%**	**33.8%**

As for *Pseudomonas aeruginosa*, there was no change in resistance to nitrofurantoin after curing as all strains remained resistant. There was 25% change in resistance to ciprocin after curing. There were 20%, 50%, 50%, 50% and 75% reduction in resistance to tetracycline, pefloxacin, ofloxacin, gentamycin and ampicillin respectively after curing. There were however no resistance reduction (changes) for nitrofurantoin and chloramphenicol (Table 4). On the average, there was 33.8% resistance reduction of *Pseudomonas aeruginosa* strains to all the antibiotics used.

The fragmented (cut) DNA sizes as mediated by EcoRI and Hind III restriction enzymes are shown in Table 5. All the three multidrug resistant strains of *Staph. aureus* showed one plasmid DNA each (after plasmid DNA isolation). While two *Pseudomonas aeruginosa* strains showed two plasmid DNA each, the other two showed one each (Table 5). EcoRI and Hind III restriction enzymes made one cut (one fragment) each on the three strains of *Staph. aureus* separately. The same restriction enzymes made three and two DNA cuts respectively on *Pseudomonas aeruginosa* strain 11. Similarly, the enzymes made four and two cuts respectively on *Pseudomonas aeruginosa* strain 12 and one cut apiece on strains 13 and 14. The plasmid DNA length of 4.0kb for *Staphylococcus aureus* strain 04 using the fragment size as mediated by restriction enzyme EcoRI for example was obtained by adding the antilog of the log of the DNA molecular marker weight (MMW) i.e

**Table 5:** Plasmid DNA fragment sizes after digestion with EcoRI and Hind III Endonucleases

Code	Organisms	Plasmid No	EcoRI fragment size/No	Hind fragment Size/No
04	*Staph. aureus*	1	4.0kb(1)	4.0kb (1)
06	*Staph. aureus*	1	4.2kb(1)	4.2kb (1)
10	*Staph. aureus*	1	3.9kb(1)	3.9kb
11	*Pseud. aeruginosa*	2	4.0kb 2.1kb 1.5kb (3)	4.0kb 4.4kb (2)
12	*Pseud. aeruginosa*	2	3.2kb, 3.0kb 1.3kb, 0.9kb (4)	4.0kb, 4.6kb (2)
13	*Pseud. aeruginosa*	1	3.9kb (1)	3.9kb (1)
14	*Pseud. aeruginosa*	1	3.8kb (1)	3.8kb (1)


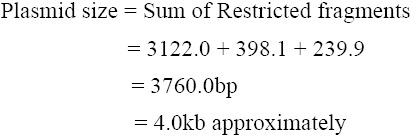


## 4. Discussion

HIV has been recovered from ocular tissues, tears and soft contact lenses of patients with AIDS (Dennehy *et al.*, 1989). All ophthalmic offices especially those with contact lens practice must be aware of any potential risk of transmission of HIV to both the office staff and other non-HIV patients through the use of trial contact lenses and tonometry (ocular examination involving contact with tears and ocular tissues).

The isolation of 36 strains made up of 27 (75.0%) *Staphylococcus aureus* and 9(25.0%) *Pseudomonas aeruginosa* from the 100 eye swab samples processed (Table 1) shows the danger these organisms may portend to all categories of opticians due to continuous exposure to them.

*Pseudomonas aeruginosa* can cause a rapidly destructive infection that can lead to loss of the entire eye (Slonim and Tampa, 1987). The organism has been found to grow better on the cornea than any other part of the eye and has also been reported to be a contaminant of many commonly used eye solutions and disinfectants (Morrison *et al.*, 1984 and Gilgardi, 1972). *Staphylococcus aureus* is the leading cause of soft tissue infections and has been implicated in eye infections (Slonim and Tampa, 1987).

All seven multidrug resistant strains before curing, recorded 85.7%, 452.9%, 14.3% and 14.3% sensitivity in that decreasing order to ciprofloxacin, pefloxacin, ofloxacin and gentamycin respectively (Table 2).

There was 0.0% sensitivity each to tetracycline and ampicillin. After curing, there was improvement in sensitivity due to removal of resistance plasmid DNA. Consequently, there was 100.0%, 85.7%, 28.6% and 71.4% improved sensitivity to the same drugs respectively. Of note, is 28.6% and 57.1% improved sensitivity to tetracycline and ampicillin after curing (Table 2). This shows or suggests that ciprofloxacin, pefloxacin, ofloxacin and gentamycin will prove to be effective in inhibiting or killing most of the strains. However after curing, ampicillin recorded 57.1% improved sensitivity as against 0.0% sensitivity before curing. However, apart from gentamycin, the others are expensive and sometimes scarce. This finding did not agree with the report of Anyanwu (1983) which stated that there is high resistance to most of the commonly used antibiotics except gentamycin. Drugs used in this study are not commonly used apart from gentamycin, tetracycline ampicillin, chloramphenicol and erythromycin. Resistance to the others apart from gentamycin and ampicillin (after curing) was high. This finding somewhat justifies the continued use of gentamycin eye drops to treat most eye infections. A combination of ampicillin and gentamycin may provide a synergistic effect to combat very debilitating eye problems.

The average resistance to all the antibiotics used on *Staphylococcus aureus* before curing was 76.2% and this reduced to 47.6% after curing suggesting a 28.6% change or reduction in resistance (Table 3). The curing of the plasmid DNA which brought about improved sensitivity is an indication that if sodium dodecyl sulphate (curing agent) is administered to the organisms in a sub lethal dose, it can lead to the elimination of plasmid DNA without adverse effect on the genomic DNA of the bacterial strains under study (Singleton and Sainsbury, 2001). A higher improvement in sensitivity to the antibiotics including nitrofurantoin and chloramphenicol was recorded for *Pseudomonas aeruginosa*. After curing, there was 33.8% reduction in resistance to the drugs on the average (Table 4).

Gel electrophoresis analysis of plasmid DNA of multidrug resistant strains after restriction enzyme digestion showed various plasmid numbers and DNA fragment sizes. The fragment sizes were used to determine overall length of plasmid DNA. For example EcoRI enzyme gave the plasmid DNA length of *Staphylococcus aureus* strain 04 as 4.0kb. The size of the DNA fragments generated by restriction enzyme cleavage depends on the distance between recognition sites (Esiobu, 2008).

## 5. Conclusion

Thirty six strains of isolates made up of 27 (75.0%) *Staphylococcus aureus* and 9 (25.0%) *Pseudomonas aeruginosa* isolated from 100 eye swabs shows the danger these organisms portend to all categories of opticians due to continuous exposure to them.

All seven multidrug resistant strains before curing, recorded 85.7%, 42.9%, 14.3% and 14.3% sensitivity in that decreasing order to ciprocin, peflacin, ofloxacin and gentamycin respectively. There was 0.0% sensitivity each to tetracycline and ampicillin. After curing, there was improvement in sensitivity as there was 100.0%, 85.7%, 28.6% and 71.4% enhanced sensitivity respectively of note, is 28.6% and 57.1% improved sensitivity to tetracycline and ampicillin after curing.

Average resistance to all antibiotics used on *Staphylococcus aureus* before curing was 76.2% which reduced to 47.6% after curing suggesting a 28.6% change or reduction in resistance. The curing of the plasmid DNA which brought about improved sensitivity is an indication that if sodium dodecyl sulphate (curing agent) is administered to the organisms in a sublethal dose, it can lead to the elimination of plasmid DNA without adverse effect on the genomic DNA of the bacterial strains under study.

Gel electrophoresis analysis of plasmid DNA of multidrug resistant strains after restriction enzyme digestion showed various plasmid Nos and DNA fragment sizes. EcoRI enzyme gave the plasmid DNA length of *Staphylococcus aureus* strain 04 as 4.0kb and this size depended upon the distance between recognition sites.
